# Supplementary material for: A Global Screen for Assembly State Changes of the Mitotic Proteome by SEC-SWATH-MS
Source: Cell Syst. 2020 Feb 26;10(2):133–155.e6. doi: 10.1016/j.cels.2020.01.001 (PMC7042714; doi:10.1016/j.cels.2020.01.001)

Q86UY8 | NT5D3_HUMAN | NT5DC3 GNN TU12B1−TY
Monomer MW [kDa]: 63.42 Monomer expected elution fraction: 44

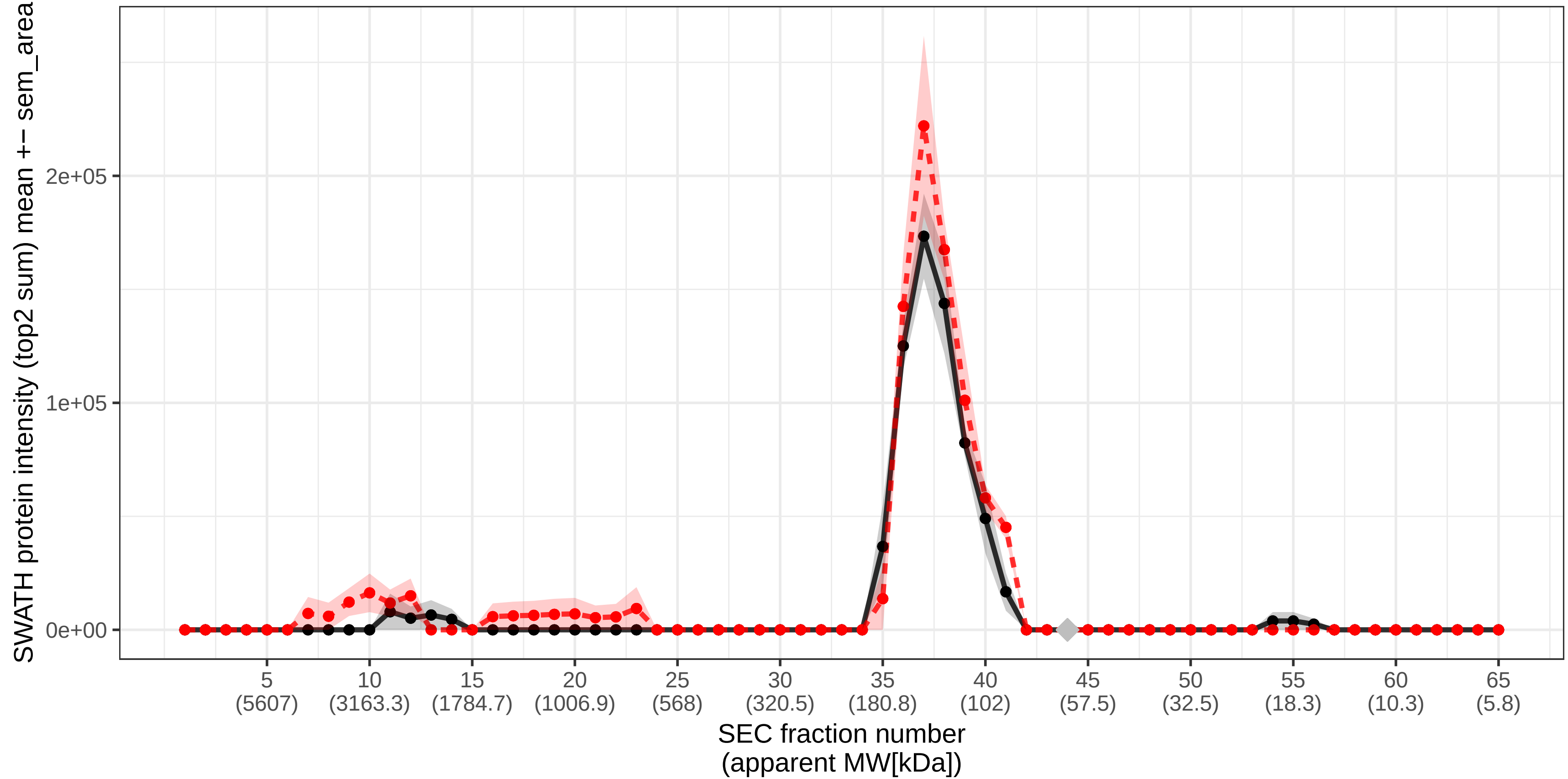

Supplement: Data S1. SEC-SWATH-MS Protein Chromatograms, Related to Figure 1 [file mmc6.zip › SECchrom_Q86UY8_NT5D3_HUMAN_NT5DC3_GNN_TU12B1-TY.pdf]
